# Fabrication and Application of Grinding Wheels with Soft and Hard Composite Structures for Silicon Carbide Substrate Precision Processing

**DOI:** 10.3390/ma17092079

**Published:** 2024-04-28

**Authors:** Qiufa Luo, Jieming Chen, Jing Lu, Congming Ke, Guangqiu Hu, Hui Huang

**Affiliations:** 1 Institute of Manufacturing Engineering, Huaqiao University, Xiamen 361021, China; jmchen1996@stu.hqu.edu.cn (J.C.); cmke@hqu.edu.cn (C.K.); huangh@hqu.edu.cn (H.H.); 2 National & Local Joint Engineering Research Center for Intelligent Manufacturing Technology of Brittle Materials Products, Xiamen 361021, China; 3 National Key Laboratory of High Performance Tools, Xiamen 361021, China; 4 Su Zhou Sail Advanced Material Co., Ltd., Suzhou 215000, China; lingdy@126.com

**Keywords:** silicon carbide, grinding wheel, soft and hard composite structure, ultra-precision processing, cutting depth in nanoscale

## Abstract

In silicon carbide processing, the surface and subsurface damage caused by fixed abrasive grinding significantly affects the allowance of the next polishing process. A novel grinding wheel with a soft and hard composite structure was fabricated for the ultra-precision processing of SiC substrates, and the grinding performance of the grinding wheel was assessed in this study. Different types of gels, heating temperatures, and composition ratios were used to fabricate the grinding wheel. The grinding performance of the grinding wheel was investigated based on the surface integrity and subsurface damage of SiC substrates. The results showed that the grinding wheel with a soft and hard composite structure was successfully fabricated using freeze-dried gel with a heating temperature of 110 °C, and the component ratio of resin to gel was 4:6. A smooth SiC substrate surface with almost no cracks was obtained after processing with the grinding wheel. The abrasive exposure height was controlled by manipulating the type and ratio of the gel. Furthermore, the cutting depth in nanoscale could be achieved by controlling the abrasive exposure height. Therefore, the fabrication and application of the grinding wheels with soft and hard composite structures is important for the ultra-precision processing of large-size SiC substrates.

## 1. Introduction

Silicon carbide (SiC) is a third-generation semiconductor material known for its superior thermal and electrical properties, such as a wide bandwidth, high thermal conductivity, high-temperature stability, and low dielectric constant, compared with those of the first- and second-generation semiconductor materials. Owing to these advantages, SiC has been widely used under high-temperature, high-frequency, high-power, and radiation-resistant working conditions [1,2,3,4]. In the manufacturing of high-performance microelectronic and optoelectronic devices, the quality that requires sub-nanometre roughness on the surface while avoiding any damage to the surface and subsurface of the SiC substrate is crucial for device performance [5,6,7]. Self-rotating grinding is one of the most effective methods for processing large SiC. The aim of self-rotation grinding is to rapidly remove cutting marks, control surface quality, and reduce the allowance for ultraprecision polishing [8,9,10]. However, owing to its hardness and brittleness, SiC is regarded as a difficult-to-process material. This presents significant challenges to the grinding process, including grinding damage and surface integrity [11,12,13].

Researchers have conducted numerous studies in the field of the SiC grinding process, ranging from conventional grinding methods to more recent nano-grinding techniques [14,15,16,17]. However, the high hardness and chemical stability of SiC pose challenges in achieving a balance between processing efficiency and quality. Various novel machining methods, such as laser-assisted grinding [18,19,20], ultrasonic vibration-assisted grinding [21,22,23], and electric discharge grinding [24,25,26], have been developed and applied to SiC substrate processing. Luo et al. [18] used laser-assisted grinding on SiC and achieved a reduction of 37–40% in the surface roughness compared with that by traditional grinding. Yan et al. [27] used electrical discharge grinding on SiC and obtained a surface roughness of 1.85 nm. Although some beneficial effects have been achieved, the amplification effect of a large size superimposed on material characteristics that are difficult to process presents significant challenges to these new methods.

Nanometre surface roughness can be obtained by nano-grinding that is considered an effective method for large-size SiC ultra-precision processing. However, the non-uniform dispersion of ultra-fine abrasives leads to the agglomeration of abrasives that causes an uneven exposure height of abrasives. This ultimately results in the formation of deep submicron cracks. Therefore, researchers have further optimised the fabrication process of ultra-fine abrasive grinding wheels. For example, Ding et al. [28] developed a porous metal-bonded cubic boron nitride (CBN) grinding wheel with significantly improved bending strength. Miao et al. [29] successfully prepared a vitrified–bonded ultra-fine diamond grinding wheel using gel casting and a pore-forming agent, and an approximately 5.0 nm roughness and a 0.21 μm damaged layer of Si surface could be obtained after processing by the grinding wheel. Feng et al. [30] prepared a PVA/PF composite sol-gel diamond grinding wheel that achieved a surface roughness of 6.42 nm for machined SiC wafers. These researchers achieved certain results; however, evident cracks and serious subsurface damage remained after processing.

In this study, a technique that used biopolymers for tool preparation, named the sol-gel technology [31,32], was used to fabricate a semi-fixed abrasive grinding wheel with a soft and hard composite structure. The biopolymers have a fast cross-linking reaction with divalent metal ions (such as Ca^2+^) and then form an ordered structure gel. Compared with a fixed abrasive grinding wheel, the use of this new grinding wheel can reduce or even eliminate the subsurface damage of SiC substrates. Different types of gels, heating temperatures, and composition ratios were explored to fabricate the grinding wheel. The performance of the grinding wheel was evaluated by analysing the surface roughness and subsurface damage of the SiC substrate.

## 2. Experimental

### 2.1. Fabrication of the Grinding Wheel

The most commonly used bonding agents for the fabrication of grinding wheels are metals, ceramics, and resins. Compared with the other two types, resin bonding agents have a lower hardness and stronger viscosity that enables them to act as effective binders. Generally speaking, the classic grinding wheels used for self-rotation grinding have a hard construction, whereas the polishing pads used for polishing have a soft construction. A novel grinding wheel with a unique soft and hard composite structure was fabricated by selecting resin as the hard binder and gel as the soft binder according to the design concept of overall hardness with partial softness. Different gel types were purchased from Suzhou Sail New Materials Co., Ltd. (Suzhou, China).

The grinding wheel was fabricated in five steps. First, the sodium alginate gel with 100% concentration W10 diamond abrasives was crushed and dispersed into a powder ranging in size from 0.2 mm to 0.5 mm, and the resin and gel were mixed for 2 h to ensure better blending. Second, 0.498 g of the mixed powder was weighed using a precise electronic balance for each block. A graphite mould measuring 19 mm × 3 mm × 5 mm was filled with the mixed powder. Third, the filled mould was pressed for 30 s at a pressure of 10 kPa. Fourth, the blocks were heated in an electric blast drying oven, the heating temperature was set at 110 °C with a holding time of 2 h, and the heating rate was maintained at 5 °C/min. Finally, the 36 blocks were bonded to the base of the grinding wheel using epoxy resin. After drying and correction, a grinding wheel with a soft and hard composite structure was fabricated. The surface morphology of the blocks was examined using scanning electron microscopy (SEM) (ProX, Phenom, Eindhoven, The Netherlands).

### 2.2. Grinding Setup

**Figure 1:**
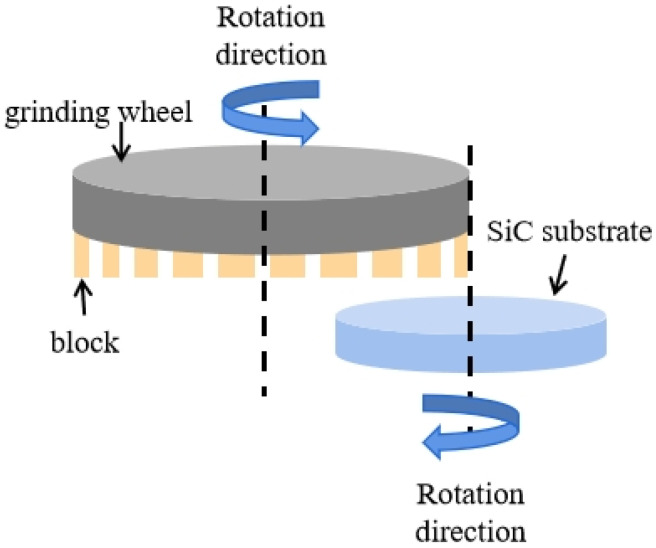
Principle of self-rotation grinding.

The grinding experiment was conducted using a self-rotation grinding machine (HRG 300, ACCRETECH, Tokyo, Japan) with the fabricated grinding wheel having a soft and hard composite structure; the principle of self-rotation grinding, as illustrated in Figure 1, was employed in this study. The SiC substrates (with a diameter of 101.6 mm) with an initial surface roughness of approximately 25 nm were used for the machining process. The C face of the SiC substrate was subjected to grinding using the grinding wheel, with a grinding wheel rotation of 1000 rpm, a feed speed of 0.3 μm/s, and a work spindle rotation of 301 rpm. In Experiment A, an ordinary fixed grinding wheel was used to process the SiC substrate. In Experiment B, the grinding wheel with the soft and hard composite structure was used. After grinding, the SiC substrate was rinsed with distilled water and dried using an air gun. The surface roughness of the SiC substrate was measured periodically using a 3D optical surface profiler (NewView 7300, ZYGO, Middlefield, OH, USA) with a scanning area of 140 µm × 105 µm. The exposure heights of the diamond abrasives on the block surfaces were observed before and after processing using a laser confocal microscope (LSM 700, Zeiss, Oberkochen, Germany). Six blocks evenly distributed on the grinding wheel were selected to observe the abrasive exposure height differences. In each block, six points were selected for the measurement, and three abrasives at each point were selected to observe the exposure height variation. The exposure height of the abrasive at each point was recorded, and the standard deviations of the three abrasive exposure heights were calculated. A cross-sectional transmission electron microscopy (TEM) specimen was prepared using a focused ion beam (FIB) (Helios 450S, FEI, Hillsboro, OR, USA). Subsurface damage to the SiC substrate was investigated using high-resolution TEM (TitanThemis 200, FEI, Hillsboro, OR, USA).

## 3. Results

### 3.1. Preparation and Mechanical Performance Characterisation of Pure Resin Blocks

**Figure 2:**
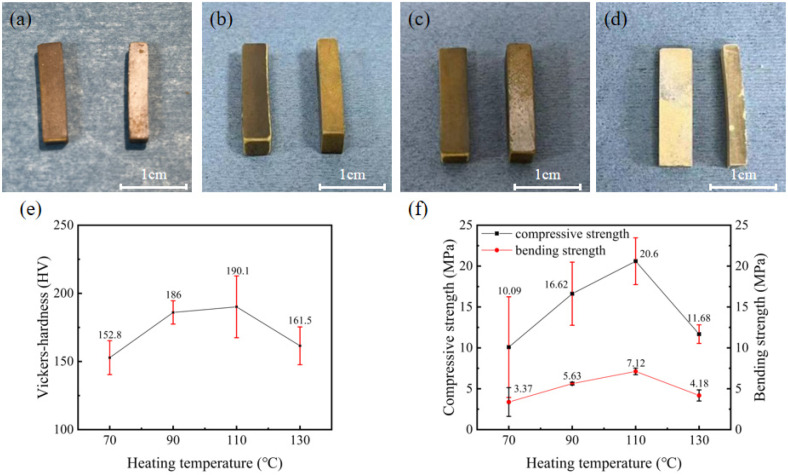
The morphological features of blocks with different heating temperatures and their mechanical properties: (**a**) 70 °C; (**b**) 90 °C; (**c**) 110 °C; (**d**) 130 °C; (**e**,**f**) represent their hardness and compressive strength and bending strength.

Different pure resin blocks were produced by varying the heating temperature. The pure resin blocks prepared at heating temperatures of 70 °C, 90 °C, 110 °C and 130 °C are illustrated in Figure 2a–d, respectively. As evidently shown in the figure, as the heating temperature increased, the colour of the pure resin blocks deepened and turned yellow, which meant the resin was more fully heated. When the temperature was elevated from 70 °C to 110 °C, the change in the shape of the blocks was minimal, and they all had a good shape retention ability. However, with a heating temperature of 130 °C, the edges of the blocks exhibited slight signs of melting owing to the excessive temperature.

A microhardness tester and a universal testing machine were used to measure the hardness, compressive strength, and bending strength of the blocks. The hardness, compressive strength, and bending strength of pure resin blocks at different heating temperatures are shown in Figure 2e,f. As observed from the figures, at a heating temperature of 70 °C, the hardness of the blocks was 152.8 HV, the compressive strength was 10.09 MPa, and the bending strength was 3.37 MPa. The hardness, compressive strength, and bending strength of the blocks at a heating temperature of 90 °C were 186.0 HV, 16.62 MPa, and 5.63 MPa, respectively. When the heating temperature was 110 °C, the values for hardness, compressive strength, and bending strength of the blocks were 190.1 HV, 20.60 MPa, and 7.12 MPa, respectively. Continuously increasing the temperature to 130 °C resulted in a hardness of 161.5 HV, with a compressive strength of 11.68 MPa and bending strength of 4.18 MPa.

### 3.2. Preparation and Mechanical Performance Characterisation of Blocks with Soft and Hard Composite Structures

**Figure 3:**
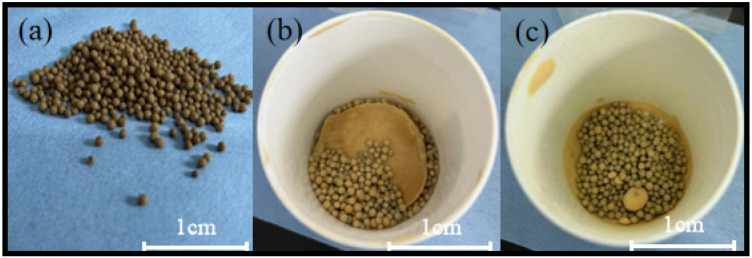
The morphological features of wet gel (**a**) and resin mixture (**b**,**c**).

Different types of gels were used to prepare the soft and hard composite structural blocks. Figure 3 illustrates the results obtained using wet gel. The morphology of the wet gel is shown in Figure 3a, whereas Figure 3b shows the resin gel mixture after 2 h of mixing, revealing solidified and bonded resins with a small portion adhering to the surface of the wet gel spheres. The resin gel mixture after 5 min of mixing is illustrated in Figure 3c; the resins remained solidified and formed clusters, with a small amount of resin powder present on the surface of the wet gel spheres.

**Figure 4:**
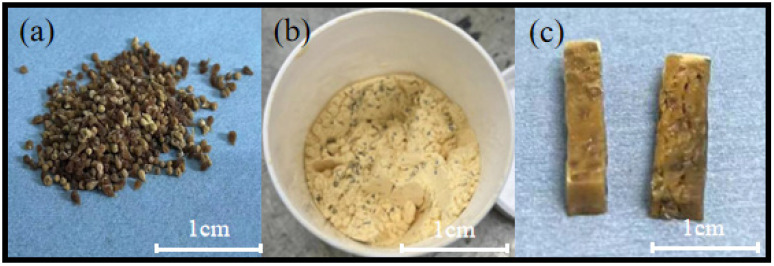
The morphological features of dry gel (**a**), resin mixture (**b**), and blocks (**c**).

The results for the blocks prepared using dry gel are presented in Figure 4. The morphology of the dry gel is shown in Figure 4a, and the resin gel mixture after 2 h of mixing is shown in Figure 4b. In this case, the resin powder did not solidify into a group and the dry gel spheres were uniformly dispersed. Additionally, Figure 4c shows the soft and hard composite structure of the blocks heated at 110 °C, revealing a dark brown colour, noticeable surface cracks, and jagged edges.

**Figure 5:**
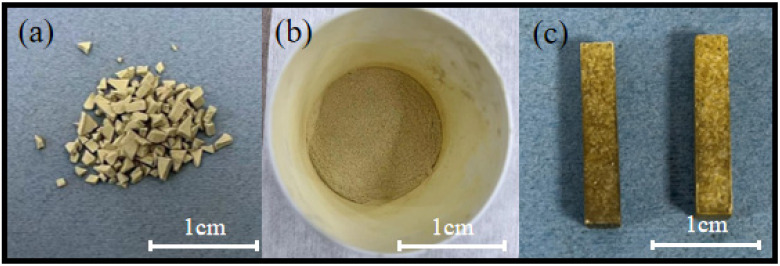
The morphological features of freeze-dried gel (**a**), resin mixture (**b**), and blocks (**c**).

The morphology of the freeze-dried gel blocks used for block preparation is shown in Figure 5a. The mixture after shearing and crushing of the freeze-dried gel powder mixed with the resin powder for 2 h is shown in Figure 5b, illustrating a uniform mix of resin powder and gel powder. Finally, the blocks of the soft and hard composite structure heated at 110 °C are shown in Figure 5c, demonstrating a light yellow colour, absence of cracks, overall density, and strong type-retaining capacity. The black colour on the surface was the result of compression in the graphite moulds.

**Figure 6:**
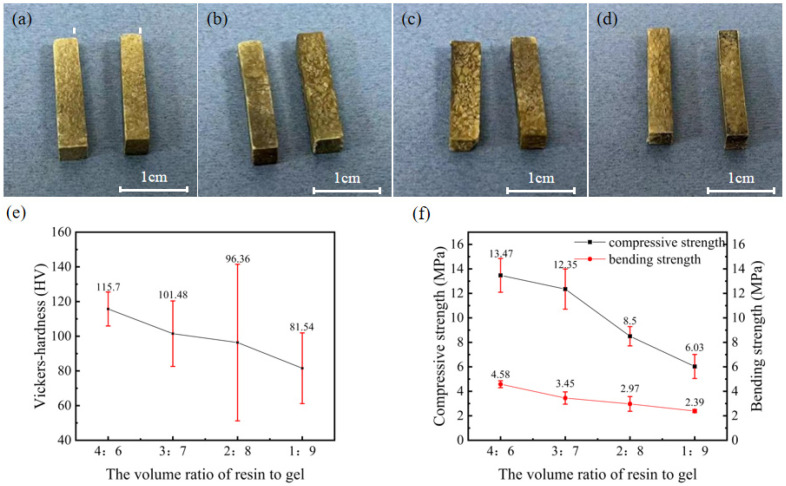
The morphological features of soft and hard composite blocks with different ratios and their mechanical properties: (**a**) resin:gel = 4:6; (**b**) resin:gel = 3:7; (**c**) resin:gel = 2:8; (**d**) resin:gel = 1:9; (**e**,**f**) represent their hardness and compressive strength and bending strength.

To vary the volume ratio of resin to gel in the soft and hard composite structural blocks, different ratios of resin to gel, that is, 4:6, 3:7, 2:8, and 1:9, were used, as shown in Figure 6a–d, respectively, at a heating temperature of 110 °C. Although the surface appearance of the prepared blocks did not significantly change with decreasing volume occupied by the resin powder, a certain amount of slagging was observed upon contact. The hardness, compressive strength, and bending strength of the soft and hard composite structures with different ratios are shown in Figure 6e,f. Notably, a decrease in the volume ratio resulted in a decrease in the hardness, compressive strength, and bending strength of the blocks. Notably, at the volume ratio of 4:6, the hardness was 115.7 HV, compressive strength was 13.47 MPa, and bending strength was 4.58 MPa. The hardness, compressive strength, and bending strength of the blocks at a ratio of 3:7 were 101.48 HV, 12.35 MPa, and 3.45 MPa, respectively. When the ratio was 2:8, the values for hardness, compressive strength, and bending strength were 96.36 HV, 8.5 MPa, and 2.97 MPa, respectively. The reduced content of resin caused an uneven distribution of resin and gel, which may have resulted in large fluctuations in the hardness at individual points. A change in the ratio to 1:9 resulted in a decrease in the hardness, compressive strength, and bending strength values to 81.54 HV, 6.03 MPa, and 2.39 MPa, respectively.

### 3.3. Grinding Performance of the Soft and Hard Composite Structure Grinding Wheel

**Figure 7:**
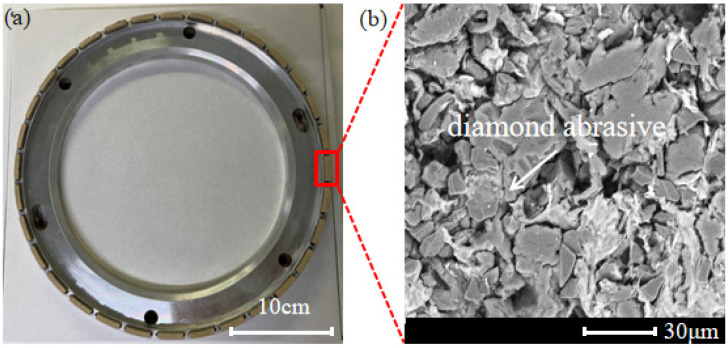
The morphological features of grinding wheel with a soft and hard composite structure (**a**) and the SEM image of the block (**b**).

Based on the aforementioned experiment, the freeze-dried gel was selected with a heating temperature of 110 °C and a resin gel ratio of 4:6 for block preparation. Enough blocks were prepared and the ones with a good morphology were selected for the preparation of the wheel. The prepared blocks measuring 19 mm × 3 mm × 5 mm were firmly attached to a stainless steel base using an epoxy resin and then trimmed. The grinding wheel with a soft and hard composite structure, as shown in Figure 7a, consisted of 32 blocks evenly distributed around the stainless steel base. Additionally, the grinding wheel featured six positioning holes for convenient installation on the HRG 300. SEM analysis was conducted to observe the surface of the blocks, and the results, as shown in Figure 7b, revealed that the resin acted as a bonding agent, and the diamond abrasives were encapsulated by the gel.

**Figure 8:**
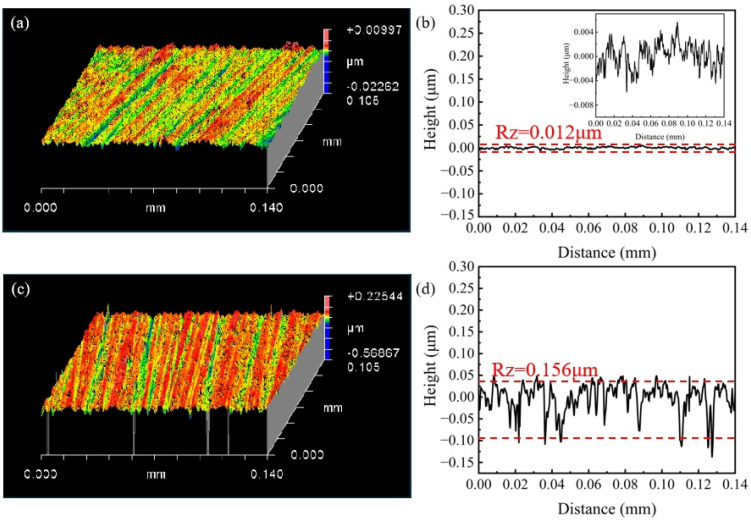
Surface topographies of SiC substrates processed by the grinding wheel with a soft and hard composite structure (**a**,**b**) and fixed abrasive grinding wheel (**c**,**d**).

Two different types of grinding wheels with diamond abrasives were used to machine the SiC substrates with a diameter of 101.6 mm. All the grinding wheels had an abrasive size of 10 μm and a concentration of 100%. The resulting surface roughness of the SiC substrate after grinding is shown in Figure 8. The surface roughness of the SiC substrate after processing with the grinding wheel with a soft and hard composite structure is shown in Figure 8a,b whereas that after processing with ordinary fixed abrasive grinding wheels is shown in Figure 8c,d. After using the grinding wheel with a soft and hard composite structure, the SiC substrate exhibited a Ra of only 1.35 nm and a Rz of 0.012 μm. Conversely, when processed with the ordinary fixed abrasive grinding wheels, the SiC substrate showed a Ra of 24.69 nm and a significantly higher Rz of 0.156 μm that surpassed the results obtained from using the grinding wheel with a soft and hard composite structure.

**Figure 9:**
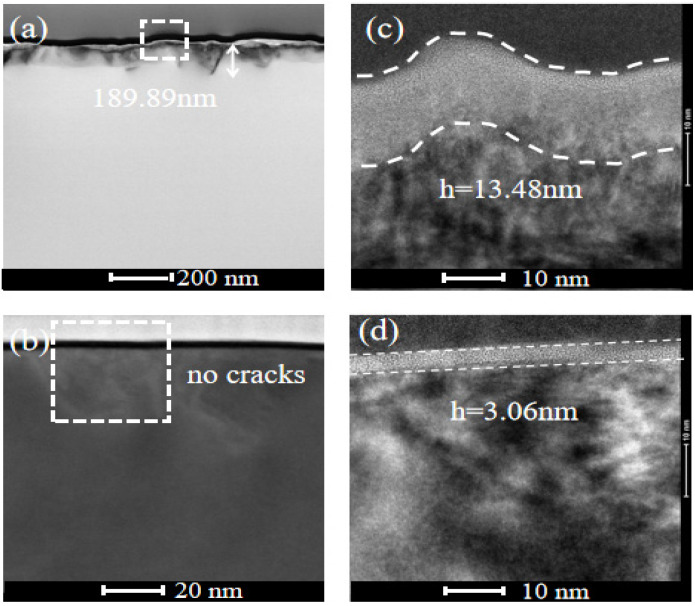
Subsurface topographies of SiC substrates processed by the fixed abrasive grinding wheel (**a**) and grinding wheel with a soft and hard composite structure (**b**). (**c**,**d**) Are acquired from the areas marked by the dashed square in (**a**,**b**), respectively.

The subsurface damage to the processed SiC was examined using projection electron microscopy, as illustrated in Figure 9. The STEM images of the SiC samples after machining with an ordinary fixed abrasive grinding wheel are presented in Figure 9a,c. These images revealed the presence of noticeable cracks, measuring up to 189.89 nm in depth, on the subsurface, along with severe dislocations and an amorphous layer of 13.48 nm. The STEM images of the SiC samples machined with the grinding wheel with a soft and hard composite structure are shown in Figure 9b,d. Notably, cracks were invisible on the subsurface, and the amorphous layer was approximately 3.06 nm.

**Figure 10:**
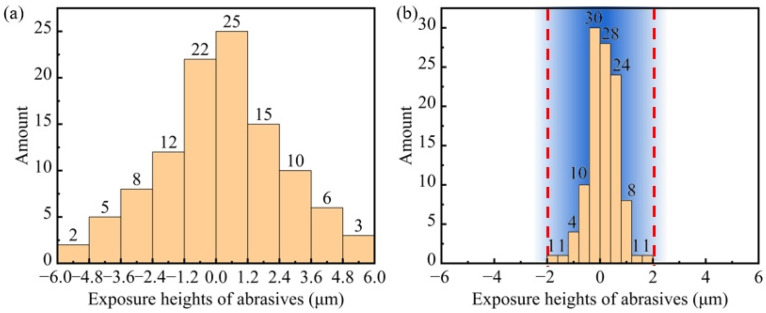
Exposure heights of abrasives before (**a**) and after (**b**) machining.

The surface abrasive exposure heights of the grinding wheel with a soft and hard composite structure were examined before and after grinding. The statistics of the exposure heights of the abrasives are shown in Figure 10. The exposure heights of the abrasives on the surfaces of the blocks before grinding are shown in Figure 10a. The exposure heights followed a normal distribution with a maximum difference of 12.0 µm. However, after grinding (Figure 10b), the exposure heights of over 85% of the abrasives were within the range of −0.8 µm to +0.8 µm, whereas that before processing were within the range of −3.6 µm to +3.6 µm. The maximum difference in the abrasive exposure height was merely 4 µm; this was significantly smaller than the value observed before grinding.

**Figure 11:**
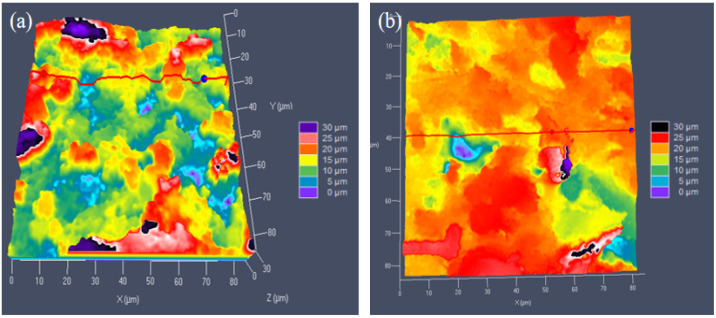
Block surface before (**a**) and after (**b**) grinding.

Figure 11a,b shows the height cloud of the block surface before and after grinding, respectively. As shown in Figure 11a, the difference between the highest and the lowest points of the block surface was significant, whereas, as shown in Figure 11b, the block surface was relatively flat. The mean value of the exposure height of the abrasives in each observation surface was calculated before and after the grinding of the block. Six blocks were considered, with six observation points on each block. In conclusion, the average of the standard deviation of the exposure height of the abrasives on the surface of the blocks before the processing of the block was 3.14, whereas that after the processing of the blocks was 0.82.

## 4. Discussion

The strength of the blocks was determined by the resin that functioned as the hard part, ensuring the strength requirements of the blocks with soft and hard composite structures under high rotation speeds. By controlling the temperature during the heating process of the blocks and analysing their mechanical properties, notably, the hardness, compressive strength, and bending strength of the blocks initially increased and then decreased as the temperature increased from 70 °C to 130 °C. Specifically, when the temperature increased from 70 °C to 110 °C, the mentioned properties continued to improve until reaching their peak, indicating the optimal comprehensive mechanical properties of the blocks at this temperature. However, when the temperature exceeded 130 °C, which surpasses the melting point of the resin, the hardness, compressive strength, and bending strength of the prepared blocks notably declined. Therefore, whereas increasing the temperature enhanced the strength of the blocks, excessively high temperatures overcooked the resin, resulting in decreased hardness, compressive strength, and bending strength. Therefore, the appropriate temperature was approximately 110 °C.

The exposure height of the abrasives and the uniform material removal were controlled by the gel that acted as the soft part of the soft and hard composite structure. To prepare the blocks, three different types of gels (wet, dry, and freeze dried) were mixed with the resin. The resin was solidified by mixing with the wet gel. The blocks could be obtained by using the dry gel; however, the cracked surfaces and low strength rendered further processing unfeasible. Blocks with higher strength could be produced only by applying the freeze-dried gel. Thus, the selection of the gel in the blocks was crucial. Only the freeze-dried gel, that ensured the absence of moisture, could be used to fabricate the blocks. The overall performances of the blocks were affected during subsequent heating processes because the dry gel contained moisture. The volume ratio of the resin to gel was modified to prepare the blocks; as the proportion of resin gradually decreased, the hardness, compressive strength, and bending strength of the blocks also decreased. At a resin volume ratio of 10%, the blocks in the loose state were easily crushed when touched. Consequently, the resin not only acted as a hard binding agent but also functioned as a binder. The processing requirements could not be satisfied because of the reduction in the resin content that led to a decrease in strength. More than half of the entire block volume should be occupied by the gel to ensure an adequate content of diamond abrasives in the blocks for efficient material removal. Therefore, a volume ratio of 40% resin to 60% gel was appropriate.

**Figure 12:**
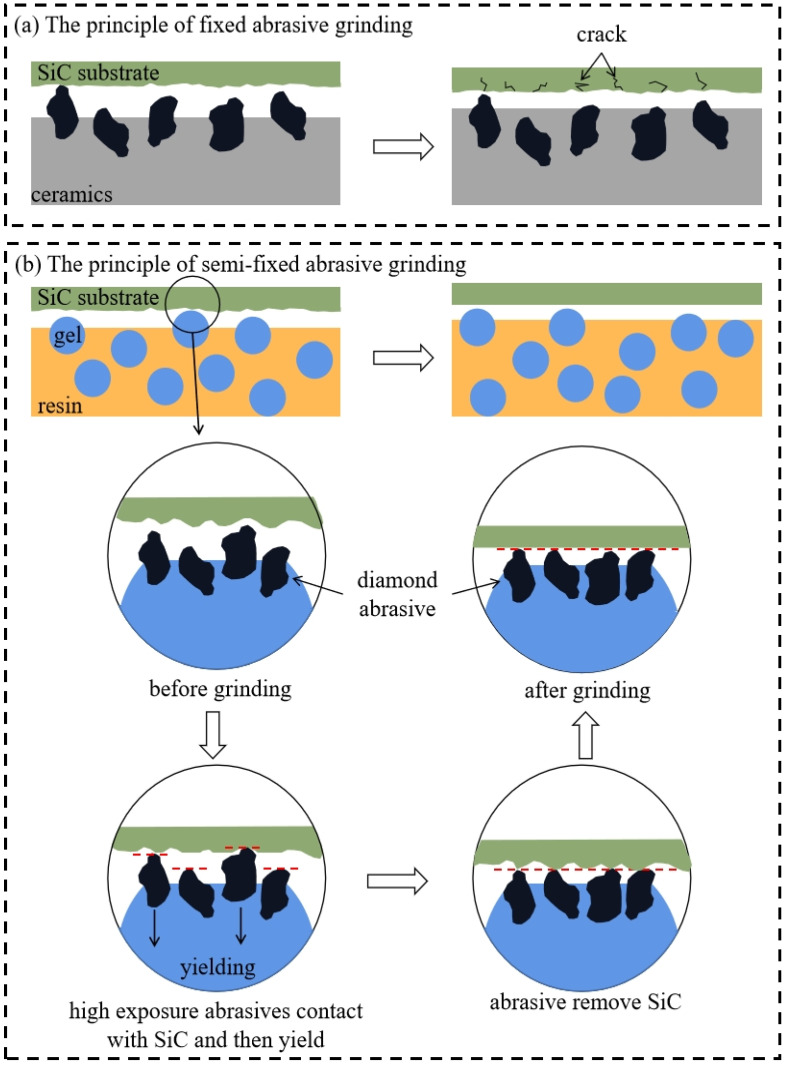
Principle of fixed (**a**) and semi-fixed (**b**) abrasive grinding.

Completely different processing results were achieved using a fixed abrasive wheel and a grinding wheel with a soft and hard composite structure for grinding the SiC substrate. SiC substrates processed by the grinding wheel with a soft and hard composite structure exhibited a lower surface roughness, fewer and shallower scratches, and less subsurface damage. The principles of fixed and semi-fixed abrasive grinding are illustrated in Figure 12. The exposure height of the abrasives could not be controlled by the fixed abrasive grinding wheel because of the strong bonding of the ceramic binder. The inability of the fixed abrasive grinding wheel to yield abrasives with a high exposure height during grinding resulted in deeper scratches and serious damage to the subsurface. However, the surface quality after processing, with a Ra as low as 1.35 nm, was significantly improved by using the grinding wheel with a soft and hard composite structure under the same conditions. The ‘overall hard, partially soft’ structure was realised through the mix of the resin and gel. The abrasives with a large exposure height were yielded to ensure that they could be machined in approximately the same horizontal plane owing to the use of soft gel when the abrasives contacted the processing surface during grinding. The exposure height of abrasives before and after machining decreased by approximately 77.8% through the results of the statistics in Figure 10. Moreover, because the abrasives were predominantly processed in the same horizontal plane, the surface was extremely smooth, with no scratches or subsurface cracks.

The exposure heights of the abrasives on the surface of the blocks before and after processing were observed by laser confocal microscopy; the difference in the abrasive exposure heights before grinding was large, whereas that after grinding was significantly reduced. This indicated that the exposure height of abrasives could be controlled using a grinding wheel with a soft and hard composite structure, and the cutting depth in nanoscale was realised by a significant reduction in the exposure height of the abrasives. Cheng et al. [33] discovered that, when the cutting depth was limited to the nanoscale, silicon dioxide (SiO_2_) could be produced by the reaction between SiC and water. By effectively controlling the yielding effect of the abrasives, the cutting depth of the abrasives at the nanoscale can be precisely controlled. This enables a reaction between SiC and water, resulting in enhanced material removal rates and improved processing quality. Thus, the investigation of the interfacial reaction at a nanoscale cutting depth are also important directions in our next research.

In conclusion, the strength of the blocks was significantly influenced by the heating temperature and ratio of resin to gel. A soft and hard composite structure that can control the exposure heights of the abrasives was realised by adding a soft binder to a hard binder. By effectively controlling the exposure height of the abrasives, the Ra was remarkably reduced, subsurface damage was minimised, and the cutting depth could be controlled at the nanoscale.

## 5. Conclusions

This study fabricated a grinding wheel with a soft and hard composite structure and investigated the influence of the heating temperature, gel type, and resin–gel ratio on the blocks. The grinding performance of the wheel was evaluated based on surface roughness and subsurface damage. The following major conclusions were drawn from this study.

1. The desired composite structure of the blocks was successfully fabricated by mixing the freeze-dried gel with the resin. When the heating temperature was 110 °C and the volume ratio of resin to gel was 4:6, the best values for hardness, compressive strength, and bending strength were 115.7 HV, 13.47 MPa, and 4.58 MPa, respectively. The mechanical performance characterisation was primarily influenced by the heating temperature and ratio of the resin to gel.
2. Upon processing the SiC substrate using a fixed abrasive grinding wheel, the resulting roughness was measured at 24.69 nm, with cracks reaching a depth of 189.89 nm. In contrast, after using the grinding wheel with a soft and hard composite structure, the SiC substrate exhibited a significantly improved roughness of only 1.35 nm. Furthermore, a thin amorphous layer measuring 3.06 nm and devoid of cracks was observed on the subsurface.
3. The use of soft gel during processing exerted pressure on the abrasives, causing them to yield into the blocks, reducing the exposure height of the abrasives, and enabling material removal on the same horizontal plane. The nanoscale cutting depth could be controlled by controlling the yielding effect of the abrasives.

## Data Availability

The data presented in this study are available on request from the corresponding author.
